# Artificial Intelligence for COVID-19: Rapid Review

**DOI:** 10.2196/21476

**Published:** 2020-10-27

**Authors:** Jiayang Chen, Kay Choong See

**Affiliations:** 1 Yong Loo Lin School of Medicine National University of Singapore Singapore Singapore; 2 Division of Respiratory & Critical Care Medicine Department of Medicine National University Hospital Singapore Singapore

**Keywords:** coronavirus, deep learning, machine learning, medical informatics, computing, SARS virus, COVID-19, artificial intelligence, review

## Abstract

**Background:**

COVID-19 was first discovered in December 2019 and has since evolved into a pandemic.

**Objective:**

To address this global health crisis, artificial intelligence (AI) has been deployed at various levels of the health care system. However, AI has both potential benefits and limitations. We therefore conducted a review of AI applications for COVID-19.

**Methods:**

We performed an extensive search of the PubMed and EMBASE databases for COVID-19–related English-language studies published between December 1, 2019, and March 31, 2020. We supplemented the database search with reference list checks. A thematic analysis and narrative review of AI applications for COVID-19 was conducted.

**Results:**

In total, 11 papers were included for review. AI was applied to COVID-19 in four areas: diagnosis, public health, clinical decision making, and therapeutics. We identified several limitations including insufficient data, omission of multimodal methods of AI-based assessment, delay in realization of benefits, poor internal/external validation, inability to be used by laypersons, inability to be used in resource-poor settings, presence of ethical pitfalls, and presence of legal barriers. AI could potentially be explored in four other areas: surveillance, combination with big data, operation of other core clinical services, and management of patients with COVID-19.

**Conclusions:**

In view of the continuing increase in the number of cases, and given that multiple waves of infections may occur, there is a need for effective methods to help control the COVID-19 pandemic. Despite its shortcomings, AI holds the potential to greatly augment existing human efforts, which may otherwise be overwhelmed by high patient numbers.

## Introduction

COVID-19, caused by SARS-CoV-2 [1], was first discovered in December 2019 and has since become a global pandemic [2]*.* An emerging viral pandemic like COVID-19 exerts significant pressure on limited health care resources [3]. To prevent human efforts of disease containment from being overwhelmed, we need tools that can streamline the diagnosis, surveillance, and treatment of COVID-19 [4]. This need is particularly pressing in relatively resource-scarce settings, such as low- or middle-income countries [5,6].

Digital methods such as artificial intelligence (AI) hold the potential to greatly enhance medical care [7]. AI implies the use of a computer to model intelligent behavior without human intervention [8]. It has been applied to many areas of medicine [9], especially to aid the detection and prevention of disease [10]. AI techniques being used in medicine are broad, ranging from computer vision to deep learning techniques [11]. Unlike the if-then rules used in traditional computer programming, AI methods emulate the decision-making process of humans via two major approaches. The first major approach is supervised machine learning, which aims to develop a predictive algorithm using regression (linear or multiple) or classification methods (eg, decision trees, neural networks). The other major AI approach is unsupervised machine learning, which allows computers to explore large amounts of unclassified data and to discover novel disease or treatment patterns [12].

An example of how AI has been applied to COVID-19 is demonstrated by BlueDot, a Canadian company specializing in infectious disease forecasting [13]. Using an AI engine that continuously gathers data for a multitude of diseases from a range of different sources globally, BlueDot was able to predict the COVID-19 outbreak and alert its users even before the World Health Organization did [14]. Another example is an AI-powered chatbot named SGDormBot, which has been used for symptom-based mass screening of migrant workers for COVID-19 in Singapore [15].

Nonetheless, while AI has been promoted as a tool to help manage the COVID-19 pandemic, AI has both potential benefits and limitations. We therefore conducted a rapid review of AI applications for COVID-19. In our review, we sought to delineate the major categories of AI use, describe the limitations of AI, and identify areas for further development.

## Methods

We based our review on the PRISMA (Preferred Reporting Items for Systematic Reviews and Meta-Analyses) statement [16], and extensively searched two databases (PubMed and EMBASE) for all English-language papers published from December 1, 2019, to March 31, 2020, using the search terms “novel coronavirus,” “2019 novel coronavirus,” “2019-nCoV,” “coronavirus disease 2019,” “COVID-19,” and “SARS-CoV-2.” The database search was supplemented by reference list checks. Papers reporting new data on AI applications for COVID-19 were included. Review papers and commentaries without new data were excluded.

The quality of included studies was assessed using a modified TRIPOD (transparent reporting of a multivariable prediction model for individual prognosis or diagnosis) statement for adherence to reporting standards [17] and PROBAST (prediction model risk of bias assessment tool) for risk of bias [18]. A thematic analysis and narrative review of AI applications for COVID-19 was then conducted.

## Results

### Included Studies, Adherence to Reporting Standards, and Risk of Bias

**Figure 1:**
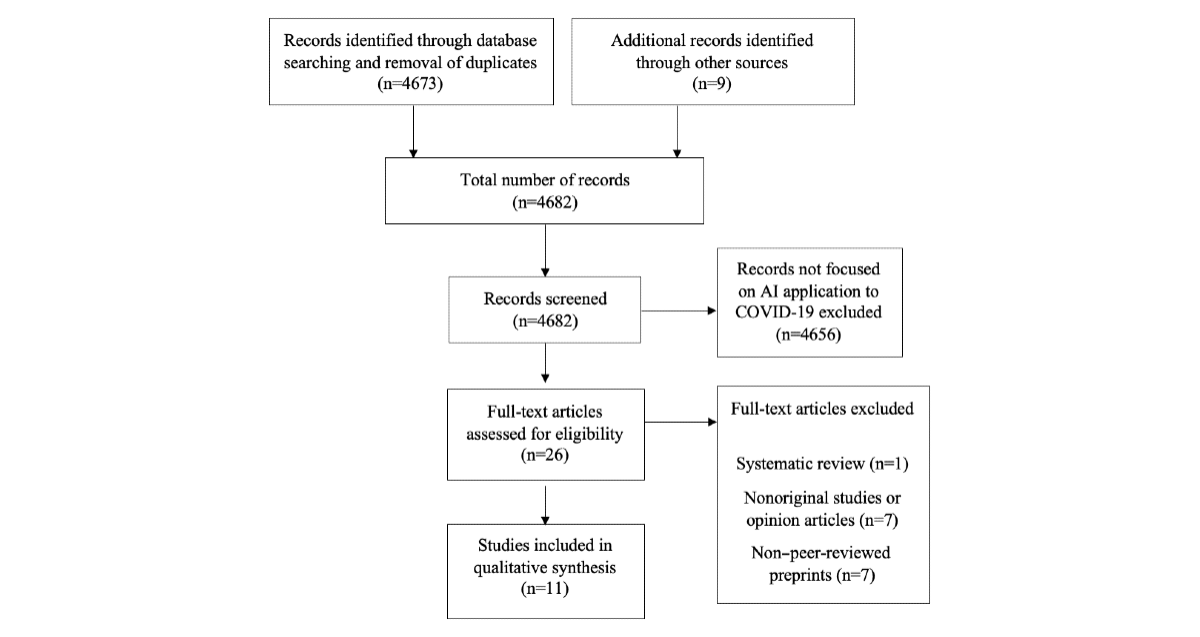
Study flow diagram.

**Figure 2:**
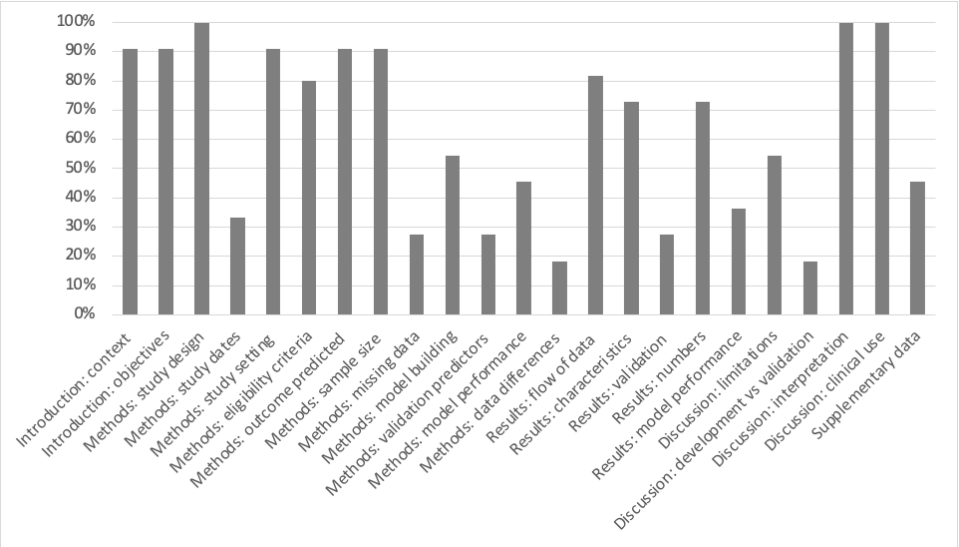
Adherence of studies to reporting standards.

**Figure 3:**
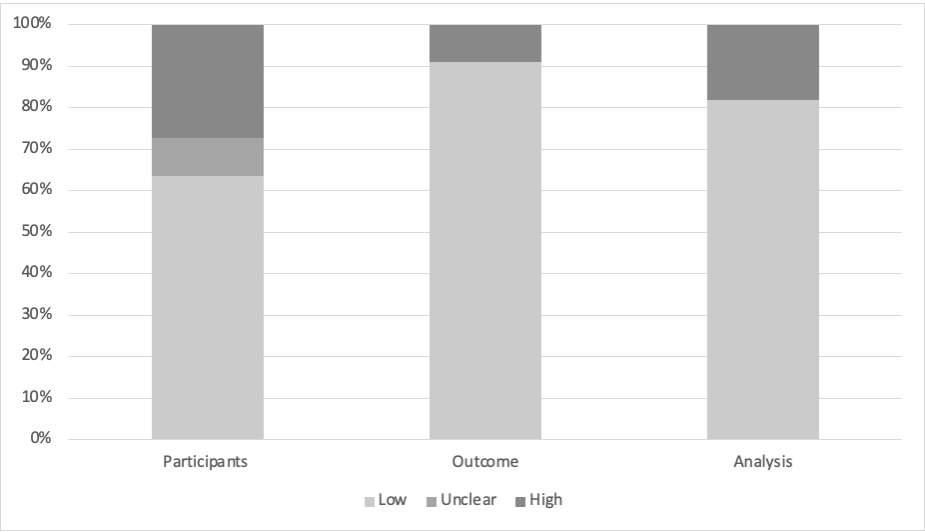
Risk of bias and applicability assessment using PROBAST (Prediction model Risk Of Bias ASsessment Tool).

Of 4682 articles, 11 articles were included for review (Figure 1, Table 1). The original TRIPOD statement consisted of a 22-category checklist with 37 items. However, items related to predictor variables were not relevant for studies assessing the performance of AI algorithms. The final components selected, as well as the degree of adherence in each category, are shown in Figure 2. Overall, adherence rates of publications to TRIPOD items ranged from 18.2% to 100%. Items concerned with data validation were reported in <50% of the 11 publications reviewed. Items related to clinical context, study methodology, and applicability were better reported, with >80% of the publications providing adequate information. For the assessment of risk of bias and applicability, we applied the PROBAST tool (Figure 3); as with TRIPOD, we did not assess predictors. Generally, risk of bias in the remaining three categories was low. We identified four areas where AI was applied to COVID-19: diagnosis, public health, clinical decision making, and therapeutics.

**Table 1:** Studies included in the review.

First author (year)	Area or specialty of AI^a^ application	AI application method	Clinical benefit shown	Internal or external validation done
Hurt (2020) [19]	Diagnosis and clinical decision making	Using a deep learning approach to augment radiographs with color probability	Improved diagnostic accuracy of pneumonia and COVID-19 at point of care, triaged patient for CT^b^ scan, helped physicians track evolution of pulmonary manifestation over length of hospitalization	No
Li (2020) [20]	Diagnosis	Deep learning–based computer-aided diagnostic system for pneumonia trained with CT scans of patients with COVID-19 suggested pneumonia in patients who received a negative reverse-transcription polymerase chain reaction test result	Improved accuracy of diagnosis	No
Li (2020) [21]	Diagnosis	AI 3D deep learning model to analyze CT scan	Improved diagnostic accuracy and differentiated from non–COVID-19 lung pathologies	Yes
Yang (2020) [22]	Public health	Recurrent neural network for AI-based prediction of epidemic trend	Good epidemiological modeling and prediction of trends relating to COVID-19	Yes
Al-Najjar (2020) [23]	Public health	AI-based classifier prediction model to determine the outcome of patients	Identified key factors influencing clinical outcome, guided public health decision making	No
Jiang (2020) [24]	Clinical decision making	Tool with AI capabilities that will predict patients at risk for more severe illnesses based on clinical parameters	AI tool predicted patients at risk for more severe illness on initial presentation, provided clinical decision support	Yes
Beck (2020) [25]	Therapeutics	Used pretrained deep learning–based system to identify commercially available drugs that could act on the viral proteins of SARS-CoV-2	Used AI to discover that atazanavir, an antiretroviral medication, is the best chemical compound due to its high inhibitory potency, among several other antiviral agents that could be used in the treatment of SARS-CoV-2	Yes
Kadioglu (2020) [26]	Therapeutics	AI combined with molecular docking to identify candidates suitable for drug repurposing via in silico methods	Supervised machine learning was used to study drug likeliness of candidate compounds, helped with evaluation of the potential of various agents	Yes
Richardson (2020) [27]	Therapeutics	Use of BenevolentAI's knowledge graph to search for approved drugs that can help treat COVID-19	Baricitinib was identified as a viable drug with tolerable side effects and potential therapeutic use in patients with COVID-19	No
Ton (2020) [28]	Therapeutics	Use of Deep Docking for accelerated screening of large chemical libraries for potential drugs against COVID-19	Screened through 1.3 billion compounds from the ZINC15 library to identify the top 1000 potential ligands against the main protease (Mpro) of SARS-CoV-2 and made them publicly available	Yes
Zhang (2020) [29]	Therapeutics	Use of AI-based dock analysis to determine whether the compounds listed in Traditional Chinese Medicine databases had potential for direct SARS-CoV-2 protein interaction	Identified 26 herbal plants containing compounds potentially active against SARS-CoV-2	No

^a^AI: artificial intelligence.

^b^CT: computed tomography.

### Diagnosis

Radiological diagnosis relies on imaging data and is amenable to deep-learning techniques [30]. For COVID-19, features seen on computed tomography (CT) images include bilateral ground-glass opacification and consolidation [31]. One fully automatic 3D deep-learning framework developed for the detection of COVID-19 (COVNet) was able to extract relevant information from both 2D and 3D images obtained from a CT scan to generate a probability score to distinguish patients with COVID-19 from patients with non–COVID-19 community-acquired pneumonia [21]. Once trained, COVNet could process each CT scan with high sensitivity (90%) and specificity (96%) for COVID-19 identification. In contrast to CT, real-time reverse-transcription polymerase chain reaction (RT-PCR), a technique frequently used for COVID-19 diagnosis, had a much lower sensitivity (71%-80%) [20,32]. Another AI system (InferRead CT Pneumonia), a deep learning diagnostic system, was able to identify features of coronavirus infection in CT scans of patients with false-negative RT-PCR results [33]. Even without CT images, using only plain chest radiographs, a deep learning approach could improve diagnosis of COVID-19 pneumonia by augmenting radiographs with color probability [19].

Apart from superior sensitivity compared to RT-PCR, AI coupled with radiological investigations could be more efficient for COVID-19 diagnosis. For instance, COVNet could process each CT scan in under 5 seconds on average [21]. This could both decrease physician workload and allow a greater volume of patients to be evaluated, facilitating more rapid case detection [34].

### Public Health

AI has proven to be a valuable tool in public health efforts, helping to characterize the epidemiology of COVID-19 and model disease transmission, even in the early stages of the pandemic. The application of AI to this area would be particularly helpful for policy makers, in contrast to clinical decision making by individual clinicians.

Perhaps one of the most prominent examples of AI used in public health, BlueDot has demonstrated its effectiveness in predicting and monitoring infectious diseases like COVID-19. Other applications of AI gathered from our rapid review are discussed as follows. Yang et al [22] developed an AI-based model that used a form of recurrent neural network (RNN) for epidemiological modelling. The model was trained using the 2003 severe acute respiratory syndrome (SARS) epidemic data from China, while incorporating the epidemiological parameters of COVID-19 and public health interventions like the lockdown of Hubei province. Using the model, it was predicted that the number of cases in Hubei would peak in early February. When the number of predicted infections was plotted against real-time epidemiologic trends, there was considerable similarity between the actual numbers and predictions made by the AI, supporting the accuracy of such models for forecasting disease development. Similar work is in progress using a wide range of data sources.

An artificial neural network has also been used to build a classifier prediction model for patient outcomes in South Korea [23]. The Korea Centers for Disease Control and Prevention collected patient characteristics like age and gender, extracted the independent predictors, and classified patients with COVID-19 into 2 groups: deceased and recovered. This helped policy makers target the most vulnerable patients, directing attention and resources toward their care.

### Clinical Decision Making

AI can assist in clinical decision making regarding patients with COVID-19, including triage decisions for the optimal use of limited health care resources [24]. Hurt et al [19] used an AI-augmented system for plain chest radiographs to track and predict the pulmonary progression of COVID-19 among hospitalized patients, which helped to identify patients that needed critical care. Early identification of at-risk cases could further guide clinicians toward earlier intervention, which may result in improved outcomes. For instance, Jiang et al [24] took multiple clinical parameters and constructed an AI framework that could predict deterioration even at initial presentation, with superior accuracy compared to logistic regression.

### Therapeutics

In silico screening with AI can help identify potentially effective therapeutic agents among existing drugs (ie, drug repurposing). Deep learning technology has been used to screen 1.3 billion compounds from the ZINC15 library for drug repurposing [28]. Molecule transformer-drug target interaction (MT-DTI) was a natural language processing (NLP) tool used to predict binding affinity values between commercially available antiviral drugs and target proteins on SARS-CoV-2. This led to the identification of atazanavir, an antiretroviral medication, which may also be effective against SARS-CoV-2 [25]. Similarly, using BenevolentAI’s knowledge graph and a library of structured medical information, machine learning uncovered baricitinib, a Janus kinase inhibitor used for rheumatoid arthritis, as a safe candidate drug that could inhibit SARS-CoV-2 viral entry [27]. In another study, a similar process of virtual drug screening identified antiviral agents against hepatitis C as drugs that had high binding affinities to target proteins on SARS-CoV-2 [26].

Apart from the identification of conventional Western medications that may be applied to COVID-19, in silico screening with AI has also been applied to Chinese herbal medicines. Zhang et al [29] used molecular docking analysis to determine whether natural compounds listed in the Traditional Chinese Medicine (TCM) Systems Pharmacology Database, Encyclopedia of Traditional Chinese Medicine, and SymMap could interact directly with SARS-CoV-2 proteins. They eventually shortlisted 26 herbal plants containing potential SARS-CoV-2 antivirals for further trials.

## Discussion

### Limitations of AI Identified

Despite the many benefits that AI can bring to the table, there are some significant limitations to its use.

#### Difficult Data Collection

AI needs large amounts of training data to generate accurate predictive algorithms [35]. For instance, the European Centre for Disease Prevention and Control requires a dedicated team of epidemiologists to screen, validate, and collate data from multiple international, regional, and local sources [36]. Even data collection for smaller geographical areas is far from easy. In the city-state of Singapore, a Health Ministry–helmed national consortium has been required to assemble detailed data from various health systems, hospitals, and clinics [37].

However, even if financial and logistical resources are plentiful, large volumes of information may not be available during the early stages of disease outbreaks, which is ironically the time when prediction is most required. Hence, relying on AI early in a disease outbreak may be impractical. Nonetheless, even if large amounts of data are available, AI is not infallible, as demonstrated by the failure of GoogleFlu, a big data analysis tool for epidemiological trending for influenza [38]. Similarly, diagnostic AI tools like those applied to radiology require large volumes of both image- and non–image-based clinical data to achieve high accuracy [11].

Apart from the quantity of data, obtaining high-quality inputs can be particularly challenging. Informal data sources and news reports [39] provide heterogeneous data with inherent noise, resulting in biased results [40]. This is particularly concerning in the context of epidemiological trending and disease prediction*,* when inaccurate forecasting leads to incorrect calibration of public health responses and health care resourcing. Furthermore, the collection of data from hospitals can be complicated. Some countries, such as Singapore, achieved success with data collection efforts by having a nationwide standardized protocol and rigorous contact tracing [37]. Other countries, such as the United States, rely on individual jurisdictions to report case information to the Centers for Disease Control and Prevention (CDC), but not all jurisdictions provide daily updates. As a result, the case counts may increase at different intervals [41]. Based on a report published by the CDC COVID-19 Response Team in March, less than 6% of cases reported had information regarding the patients’ comorbidities and risk factors and this incomplete data collection has limited research efforts [42].

#### Lack of Multimodal AI Assessments

Many studies used singular data types to perform AI-driven tasks, for instance only using radiological images for the diagnosis of COVID-19 [20,21]. Assessments of patients based on a single data type may be skewed, thus highlighting the need for a multimodal AI framework capable of analyzing different data types [35]. As there is significant overlap in how the lungs respond to different pathological insults [33], and as radiological presentation often depends on an interplay of multiple factors [21], the full potential of CT-based AI algorithms may be better realized by including non–image-based clinical data. For now, based on recommendations by the Italian Society of Medical and Interventional Radiology, CT should be used as a screening tool only for symptomatic patients with specific indications, and the use of CT with AI for screening or as a first-line test is not supported [43].

#### Delay in Realization of Benefits

As useful as AI may be in helping to identify drugs and vaccines against COVID-19, such treatments are unlikely to be made widely available in the immediate future, precisely when such treatments are needed the most. There is considerable delay caused by rigorous medical trials required before approval can be given to drugs or vaccines, and it can take 12-18 months just to develop a vaccine [44]. Even with a vaccine on the market, supply shortage will likely be an issue in the face of massive demand and limited initial production [45].

#### Poor Internal Validation

Another issue we noticed was the poor internal validation done in some of the studies, which makes it hard to determine the clinical or incremental value of AI over conventional methods. Studies that discussed the performance of AI augmentation of CT scans [21] and plain radiographs [19] did not compare AI with radiologists’ evaluations, and did not describe data sets used for validation [33]. Furthermore, confounding factors can affect the internal validity of such studies, such as how variation in respiratory effort, image contrast, technique, and the resolution of radiological images may affect the accuracy of AI-based radiological interpretation frameworks [19]. After completion of our review, we found one study that addressed our validity concerns. Bai et al [46] compared AI with radiologists and demonstrated that AI alone had higher accuracy, sensitivity, and specificity than radiologists, whereas the performance of radiologists was enhanced when AI was used to augment their evaluations*.*

#### Poor External Validation

External validation could also be improved. Many studies were only applied to patients seen at single centers, or populations within the same geographical region [20,22,23]. This could mean that algorithms shown to be accurate for the population studied may fare less well in other settings. Increasing the diversity of data sets from different populations and demonstrating the reproducibility of AI-based algorithms in different settings would be required if we are to generalize the usage of AI tools [11].

#### Inability to Be Used by Laypersons

While the use of AI technology in the clinical context may seem simple, the underlying theory and operating mechanisms of these algorithms are often opaque to the untrained layperson, which includes health care professionals unfamiliar with AI. For example, one of the intrinsic drawbacks of deep learning is the lack of interpretability, as it is “impossible to determine what imaging features are being used to determine the output” [21]. Physicians may not trust AI to evaluate clinical scenarios, and would not be able to troubleshoot incorrect AI-based assessments [11]. To avoid hindering AI’s uptake, implementation frameworks need to be designed in a way that make AI easily operable and clearly understood by most health care professionals.

#### Inability to Be Used in Resource-Poor Settings

AI-based methods of disease detection, surveillance, and prognostication often require access to digital resources such as CT scanners, mobile phones, and internet access. Such resources may not be widely or consistently available in less developed regions [47]. Thus, the benefits of AI-based approaches may not be realized in resource-poor settings. Effort is needed to shift the reliance of AI from expensive technologies to cheaper and more readily accessible alternatives such as chest X-ray, point-of-care ultrasound, or even vital signs data alone.

#### Ethical Pitfalls

The use of AI may require access to personal information to generate trends, make predictions, and conduct assessments. Moreover, individual patient information has been placed online on multiple platforms [48]. The sharing of such information may lead to the infringement of privacy and personal rights. Although it may be acceptable to process personal data for disease containment in a pandemic, problems arise when the data are used for sinister purposes. As such, there need to be proper ethical guidelines and laws in place to govern the use of AI and big data. For example, the Australian Human Rights Commission has set out practical steps for researchers to control the use of AI [49]. Overall, health care organizations can be viewed by patients as trustworthy, but it is important to remember that the confidentiality of the data obtained from the public should be respected, and that there should be transparency in how institutions handle the data obtained [50].

A more subtle type of ethical pitfall concerns the need for human input when programming AI tools. AI’s superiority in calculation and computation does not necessarily translate into good decision making unless the machine has values that enable it to make an ethical choice, which requires input from a human programmer [51]. In other words, a particular AI program will make choices based on the programmer’s morality. This raises an ethical concern, as difficult public health decisions like resource allocation are often multifaceted and based on more than just a single set of ethical guidelines conforming to the beliefs of a single person or single group of people. On a smaller scale, the decision to rely solely on the computer to make a unilateral judgment on how or whether a patient should be treated based on the machine’s calculated benefit of treatment is also fraught with ethical concerns as there are some who believe that medical treatment should be discussed together with the patient [50]. As such, it is prudent to use AI only as a guide in decision making, rather than relying on it wholly.

Like all prediction methods, AI may unwittingly single out ethnic minority groups as being at high risk for disease. Unethical interpretation of prediction results may exacerbate ethnic tensions and lead to the stigmatization of and discrimination against specific ethnic groups. To illustrate, the US CDC has reported that African Americans and Hispanics have higher rates of hospitalization and mortality from COVID-19 than the White population [52]. Even though such a finding is likely to be due to differential access to health care [53]*,* the same finding may lead to misconceptions about race and inherent disease susceptibility.

#### Legal Barriers

Finally, there may be legal liabilities associated with adverse outcomes when human physicians use AI technologies for the care of their patients. When malpractice cases involving medical AI applications are involved, the legal system needs to be clear on which party holds the liability [30]. This is particularly worrisome given that the usability of AI, especially for the care of patients with COVID-19, is still relatively unknown due to the lack of sufficient evidence of its effectiveness over traditional methods. Consequently, medical professionals may hesitate when asked to use AI for patient management.

### Areas for Further Work

There are several other areas in which AI has shown significant promise.

#### Surveillance

Infrared thermal cameras used to screen the public for fever have been paired with AI-powered facial recognition systems to determine if individuals are wearing surgical masks [54]. A US-based computer vision startup has started offering a software that uses camera images to observe for compliance with social distancing rules [55]. With the proliferation of such technologies, it is increasingly evident that AI-based surveillance can greatly help with public health interventions that slow the spread of infection.

Blockchain technology is a digital method that can be used in conjunction with AI, and it refers to a verifiable permanent ledger system that can be used to store health care–related information [7]. The coupling of AI with blockchain for self-testing and tracking systems has been proposed for the surveillance of COVID-19. Not only can such a system of self-testing overcome supply chain limitations in resource-scarce countries and achieve a higher rate of testing [5], it also gives real-time feedback on population health and allows for risk stratification of suspect cases.

AI can also potentially predict the occurrence of disease outbreaks early on, thus giving health authorities more time to act. Effenberger et al [56] have demonstrated the effectiveness of using internet relative search volume (RSV) indices for the forecasting of COVID-19 outbreaks in different geographical areas. Maximum public interest, and therefore maximum RSV indices, preceded peaks in case numbers. This meant that trending RSV indices can help public health authorities predict and respond to local surges of COVID-19 cases.

#### Combination With Big Data

AI-based techniques like machine learning can be used to gather data from multiple sources for processing, which can provide novel insights. We have discussed how AI’s ability to analyze large amounts of data swiftly makes it possible to use the data to predict the likelihood of new outbreaks, model successful disease containment strategies, and to find the most effective treatment protocols [9]. However, there are some other areas of application that have yet to be explored. Sun et al [57] demonstrated the value of using a Chinese health care–oriented social network that streamed reports from local or national health authorities—along with several other international media outlets—for epidemiological studies of the disease; AI can be used to facilitate this collection.

AI, when paired with big data, can find new drugs to treat COVID-19. Integrated AI-based drug discovery methods for novel drug compounds, such as deep generative models, can use large data sets to train and generate new drugs with optimized chemical properties. Such methods are often more time-efficient than traditional computational methods [58].

In addition, AI combined with big data can also be used to assess the accuracy of online information available to the public. Stratifyd, a US-based data analytics company, scans social media posts, cross-references them with data from official sources, and alerts users when false information is identified [59]. It can thus be applied to the current pandemic to prevent misinformation about the disease from spreading online. AI can also be used to analyze international air travel data. By doing so, AI can track disease spread between countries, allowing for an assessment of importation risk at a particular location and the latter’s capacity to respond [60], estimation of disease spread from the epicenter of the outbreak [61], and identification of areas that may have undetected cases imported from other countries [62]*.*

#### Operation of Other Core Clinical Services

As the COVID-19 pandemic exerts pressure on health care resources, institutions have had to reduce the provision of clinical services. The American College of Surgeons, for example, has provided guidelines for the management of nonemergency operations, recommending hospitals to “thoughtfully review all scheduled elective procedures” [63]*.* In Singapore, nonurgent medical appointments have been rescheduled and staff have been redeployed to help manage patients with COVID-19 [64]. To mitigate the impact on non–COVID-19 patients, AI can be used to augment their care.

With regard to medical investigations, AI-based radiological interpretation algorithms can be applied to read scans for non–COVID-19 diseases. With regard to medical consultations, AI-based conversational chatbots can assume some of the duties of the physician, such as symptom screening and patient education. Chatbots are already being used to combat the pandemic. Symptoma, a symptom-to-disease digital health assistant using AI, has been shown to be highly accurate when screening for COVID-19 [65]. Similarly, chatbots have been used to rapidly screen health care workers for COVID-19, facilitating staff movements and minimizing nosocomial transmission [66]. By extension, AI-based chatbots can provide a platform for patients with non–COVID-19 conditions to receive medical care at a time when clinical resources are limited. These chatbots can potentially be augmented by smartwatch-based health monitors (eg, heart rate and electrocardiogram monitoring using the Apple Watch [67]).

#### Clinical Management of Patients With COVID-19

Studies have already established that AI can help guide management of patients with COVID-19 in general practice. AI can also potentially help in the management of critically ill patients with COVID-19. For instance, given the uncertainty over the optimal management of COVID-19–related acute respiratory distress syndrome (ARDS) [68], AI methods like reinforcement learning could be used to determine management choices to achieve the best possible clinical outcomes [69]. Besides therapeutics, machine learning strategies can be used for vaccine development against COVID-19. They have already been applied to SARS-CoV-2 proteomes, and nonstructural proteins were found to be potential vaccine candidates [70].

### Conclusion

AI is no longer new in the field of medicine, and many studies have explored how its potential to enhance medical care of patients could be realized. Even as we see the situation improving in some countries, others are still struggling to contain the spread of COVID-19. In the face of growing pressure on limited health care resources, the use of AI-driven techniques to aid in diagnosis, surveillance, finding therapeutics, and public health decision making may help improve the efficiency and effectiveness of human efforts to combat the pandemic. Another recently published review found that AI could contribute to the prevention and control of the spread of COVID-19 via several important approaches: detecting suspected cases, large-scale screening, monitoring, determining interactions with experimental therapies, pneumonia screening, using the Internet of Intelligent Things for data and information gathering and integration, allocating resources; making predictions, models, and simulations; and using robotics for medical quarantine [71]. We hope that our rapid review can help highlight additional areas for more robust AI applications and studies in the later phases of the ongoing pandemic.

## Abbreviations

AI: artificial intelligence
CDC: Centers for Disease Control and Prevention
CT: computed tomography
PRISMA: Preferred Reporting Items for Systematic Reviews and Meta-Analyses
PROBAST: prediction model risk of bias assessment tool
RSV: relative search volume
TCM: Traditional Chinese Medicine
TRIPOD: transparent reporting of a multivariable prediction model for individual prognosis or diagnosis

## References

[1] Zhou P, Yang X, Wang X, Hu B, Zhang L, Zhang W, Si H, Zhu Y, Li B, Huang C, Chen H, Chen J, Luo Y, Guo H, Jiang R, Liu M, Chen Y, Shen X, Wang X, Zheng X, Zhao K, Chen Q, Deng F, Liu L, Yan B, Zhan F, Wang Y, Xiao G, Shi Z (2020). A pneumonia outbreak associated with a new coronavirus of probable bat origin. Nature.

[2] Reeves JJ, Hollandsworth HM, Torriani FJ, Taplitz R, Abeles S, Tai-Seale M, Millen M, Clay BJ, Longhurst CA (2020). Rapid response to COVID-19: health informatics support for outbreak management in an academic health system. J Am Med Inform Assoc.

[3] Emanuel EJ, Persad G, Upshur R, Thome B, Parker M, Glickman A, Zhang C, Boyle C, Smith M, Phillips JP (2020). Fair Allocation of Scarce Medical Resources in the Time of Covid-19. N Engl J Med.

[4] Yassine HM, Shah Z (2020). How could artificial intelligence aid in the fight against coronavirus?. Expert Rev Anti Infect Ther.

[5] Mashamba-Thompson TP, Crayton ED (2020). Blockchain and Artificial Intelligence Technology for Novel Coronavirus Disease-19 Self-Testing. Diagnostics (Basel).

[6] Siow WT, Liew MF, Shrestha BR, Muchtar F, See KC (2020). Managing COVID-19 in resource-limited settings: critical care considerations. Crit Care.

[7] Ting DSW, Carin L, Dzau V, Wong TY (2020). Digital technology and COVID-19. Nat Med.

[8] Hamet P, Tremblay J (2017). Artificial intelligence in medicine. Metabolism.

[9] Hashimoto D, Witkowski E, Gao L, Meireles O, Rosman G (2020). Artificial Intelligence in Anesthesiology: Current Techniques, Clinical Applications, and Limitations. Anesthesiology.

[10] Long JB, Ehrenfeld JM (2020). The Role of Augmented Intelligence (AI) in Detecting and Preventing the Spread of Novel Coronavirus. J Med Syst.

[11] Kallianos K, Mongan J, Antani S, Henry T, Taylor A, Abuya J, Kohli M (2019). How far have we come? Artificial intelligence for chest radiograph interpretation. Clin Radiol.

[12] Deo RC (2015). Machine Learning in Medicine. Circulation.

[13] Bogoch II, Watts A, Thomas-Bachli A, Huber C, Kraemer MUG, Khan K (2020). Pneumonia of unknown aetiology in Wuhan, China: potential for international spread via commercial air travel. J Travel Med.

[14] Bowles J (2020). How Canadian AI start-up BlueDot spotted Coronavirus before anyone else had a clue. Diginomica.

[15] (2020). Coronavirus: Singapore chatbot helps doctors monitor migrant workers' health in real time. South China Morning Post.

[16] Moher D, Liberati A, Tetzlaff J, Altman DG, PRISMA Group (2009). Preferred reporting items for systematic reviews and meta-analyses: the PRISMA statement. BMJ.

[17] Moons KHM, Altman DG, Reitsma JB, Ioannidis JPA, Macaskill P, Steyerberg EW, Vickers AJ, Ransohoff DF, Collins GS (2015). Transparent Reporting of a multivariable prediction model for Individual Prognosis or Diagnosis (TRIPOD): explanation and elaboration. Ann Intern Med.

[18] Moons KG, Wolff RF, Riley RD, Whiting PF, Westwood M, Collins GS, Reitsma JB, Kleijnen J, Mallett S (2019). PROBAST: A Tool to Assess Risk of Bias and Applicability of Prediction Model Studies: Explanation and Elaboration. Ann Intern Med.

[19] Hurt B, Kligerman S, Hsiao A (2020). Deep Learning Localization of Pneumonia: 2019 Coronavirus (COVID-19) Outbreak. J Thorac Imaging.

[20] Li D, Wang D, Dong J, Wang N, Huang H, Xu H, Xia C (2020). False-Negative Results of Real-Time Reverse-Transcriptase Polymerase Chain Reaction for Severe Acute Respiratory Syndrome Coronavirus 2: Role of Deep-Learning-Based CT Diagnosis and Insights from Two Cases. Korean J Radiol.

[21] Li L, Qin L, Xu Z, Yin Y, Wang X, Kong B, Bai J, Lu Y, Fang Z, Song Q, Cao K, Liu D, Wang G, Xu Q, Fang X, Zhang S, Xia J, Xia J (2020). Using Artificial Intelligence to Detect COVID-19 and Community-acquired Pneumonia Based on Pulmonary CT: Evaluation of the Diagnostic Accuracy. Radiology.

[22] Yang Z, Zeng Z, Wang K, Wong S, Liang W, Zanin M, Liu P, Cao X, Gao Z, Mai Z, Liang J, Liu X, Li S, Li Y, Ye F, Guan W, Yang Y, Li F, Luo S, Xie Y, Liu B, Wang Z, Zhang S, Wang Y, Zhong N, He J (2020). Modified SEIR and AI prediction of the epidemics trend of COVID-19 in China under public health interventions. J Thorac Dis.

[23] Al-Najjar HN, Al-Rousan N (2020). A classifier prediction model to predict the status of Coronavirus COVID-19 patients in South Korea. Eur Rev Med Pharmacol Sci.

[24] Jiang X, Coffee M, Bari A (2020). Towards an Artificial Intelligence Framework for Data-Driven Prediction of Coronavirus Clinical Severity.

[25] Beck BR, Shin B, Choi Y, Park S, Kang K (2020). Predicting commercially available antiviral drugs that may act on the novel coronavirus (SARS-CoV-2) through a drug-target interaction deep learning model. Comput Struct Biotechnol J.

[26] Kadioglu O, Saeed M, Greten HJ, Efferth T (2020). Identification of novel compounds against three targets of SARS CoV-2 coronavirus by combined virtual screening and supervised machine learning. Bull World Health Organ.

[27] Richardson P, Griffin I, Tucker C, Smith D, Oechsle O, Phelan A, Rawling M, Savory E, Stebbing J (2020). Baricitinib as potential treatment for 2019-nCoV acute respiratory disease. The Lancet.

[28] Ton A, Gentile F, Hsing M, Ban F, Cherkasov A (2020). Rapid Identification of Potential Inhibitors of SARS-CoV-2 Main Protease by Deep Docking of 1.3 Billion Compounds. Mol Inform.

[29] Zhang D, Wu K, Zhang X, Deng S, Peng B (2020). In silico screening of Chinese herbal medicines with the potential to directly inhibit 2019 novel coronavirus. J Integr Med.

[30] Yu K, Beam AL, Kohane IS (2018). Artificial intelligence in healthcare. Nat Biomed Eng.

[31] Wang D, Hu B, Hu C, Zhu F, Liu X, Zhang J, Wang B, Xiang H, Cheng Z, Xiong Y, Zhao Y, Li Y, Wang X, Peng Z (2020). Clinical Characteristics of 138 Hospitalized Patients With 2019 Novel Coronavirus-Infected Pneumonia in Wuhan, China. JAMA.

[32] Fang Y, Zhang H, Xie J, Lin M, Ying L, Pang P, Ji W (2020). Sensitivity of Chest CT for COVID-19: Comparison to RT-PCR. Radiology.

[33] Li Y, Xia L (2020). Coronavirus Disease 2019 (COVID-19): Role of Chest CT in Diagnosis and Management. American Journal of Roentgenology.

[34] Ramesh A, Kambhampati C, Monson J, Drew P (2004). Artificial intelligence in medicine. Ann R Coll Surg Engl.

[35] Santosh KC (2020). AI-Driven Tools for Coronavirus Outbreak: Need of Active Learning and Cross-Population Train/Test Models on Multitudinal/Multimodal Data. J Med Syst.

[36] European Centre for Disease Prevention and Control How ECDC collects and processes COVID-19 data.

[37] Ng Y, Li Z, Chua YX, Chaw WL, Zhao Z, Er B, Pung R, Chiew CJ, Lye DC, Heng D, Lee VJ (2020). Evaluation of the Effectiveness of Surveillance and Containment Measures for the First 100 Patients with COVID-19 in Singapore - January 2-February 29, 2020. MMWR Morb Mortal Wkly Rep.

[38] Lazer D, Kennedy R, King G, Vespignani A (2014). Big data. The parable of Google Flu: traps in big data analysis. Science.

[39] Lai Y, Yeung W, Celi LA (2020). Urban Intelligence for Pandemic Response: Viewpoint. JMIR Public Health Surveill.

[40] Crawford K (2013). The hidden biases in big data. Harvard Business Review.

[41] Centers for Disease Control and Prevention (2020). About CDC COVID-19 Data.

[42] CDC COVID-19 Response Team (2020). Preliminary Estimates of the Prevalence of Selected Underlying Health Conditions Among Patients with Coronavirus Disease 2019 - United States, February 12-March 28, 2020. MMWR Morb Mortal Wkly Rep.

[43] Neri E, Miele V, Coppola F, Grassi R (2020). Use of CT and artificial intelligence in suspected or COVID-19 positive patients: statement of the Italian Society of Medical and Interventional Radiology. Radiol Med.

[44] Vanderslott S, Pollard A, Thomas T Coronavirus vaccine: here are the steps it will need to go through during development. The Conversation.

[45] Khamsi R (2020). If a coronavirus vaccine arrives, can the world make enough?. Nature.

[46] Bai HX, Wang R, Xiong Z, Hsieh B, Chang K, Halsey K, Tran TML, Choi JW, Wang D, Shi L, Mei J, Jiang X, Pan I, Zeng Q, Hu P, Li Y, Fu F, Huang RY, Sebro R, Yu Q, Atalay MK, Liao W (2020). Artificial Intelligence Augmentation of Radiologist Performance in Distinguishing COVID-19 from Pneumonia of Other Origin at Chest CT. Radiology.

[47] Maru DS, Schwarz R, Jason A, Basu S, Sharma A, Moore C (2010). Turning a blind eye: the mobilization of radiology services in resource-poor regions. Global Health.

[48] Naudé W (2020). Artificial intelligence vs COVID-19: limitations, constraints and pitfalls. AI Soc.

[49] Santow E (2020). Emerging from AI utopia. Science.

[50] McCradden MD, Baba A, Saha A, Ahmad S, Boparai K, Fadaiefard P, Cusimano MD (2020). Ethical concerns around use of artificial intelligence in health care research from the perspective of patients with meningioma, caregivers and health care providers: a qualitative study. CMAJ Open.

[51] Braga A, Logan R (2017). The Emperor of Strong AI Has No Clothes: Limits to Artificial Intelligence. Information.

[52] Centers for Disease Control and Prevention (2020). COVID-19 in Racial and Ethnic Minority Groups.

[53] Selden TM, Berdahl TA (2020). COVID-19 And Racial/Ethnic Disparities In Health Risk, Employment, And Household Composition. Health Aff (Millwood).

[54] Chun A (2020). In a time of coronavirus, China's investment in AI is paying off in a big way. South China Morning Post.

[55] Maslan C Social distancing detection for COVID-19.

[56] Effenberger M, Kronbichler A, Shin JI, Mayer G, Tilg H, Perco P (2020). Association of the COVID-19 pandemic with Internet Search Volumes: A Google Trends Analysis. Int J Infect Dis.

[57] Sun K, Chen J, Viboud C (2020). Early epidemiological analysis of the coronavirus disease 2019 outbreak based on crowdsourced data: a population-level observational study. Lancet Digit Health.

[58] Zhavoronkov A, Aladinskiy V, Zhebrak A (2020). Potential COVID-2019 3C-like Protease Inhibitors Designed Using Generative Deep Learning Approaches. ChemRxiv.

[59] Heaven WD (2020). AI could help with the next pandemic—but not with this one. MIT Technology Rev iew.

[60] Gilbert M, Pullano G, Pinotti F, Valdano E, Poletto C, Boëlle P, D'Ortenzio E, Yazdanpanah Y, Eholie SP, Altmann M, Gutierrez B, Kraemer MUG, Colizza V (2020). Preparedness and vulnerability of African countries against importations of COVID-19: a modelling study. The Lancet.

[61] Lai S, Bogoch I, Ruktanonchai N, Watts A, Lu X, Yang W, Yu H, Khan K, Tatem AJ (2020). Assessing spread risk of Wuhan novel coronavirus within and beyond China, January-April 2020: a travel network-based modelling study. medRxiv.

[62] De Salazar PM, Niehus R, Taylor A, Buckee C, Lipsitch M (2020). Using predicted imports of 2019-nCoV cases to determine locations that may not be identifying all imported cases. medRxiv.

[63] (2020). COVID-19: Recommendations for management of elective surgical procedures. American College of Surgeons.

[64] (2020). COVID-19: Essential healthcare services to continue operating, other services to be scaled down. Channel News Asia.

[65] Martin A, Nateqi J, Gruarin S, Munsch N, Abdarahmane I, Knapp B (2020). An artificial intelligence-based first-line defence against COVID-19: digitally screening citizens for risks via a chatbot. bioRxiv.

[66] Judson T, Odisho A, Young J, Bigazzi O, Steuer D, Gonzales R, Neinstein AB (2020). Implementation of a digital chatbot to screen health system employees during the COVID-19 pandemic. J Am Med Inform Assoc.

[67] Owen M (2020). Apple Watch ECG detects heart condition in German woman. Apple Insider.

[68] Gattinoni L, Chiumello D, Rossi S (2020). COVID-19 pneumonia: ARDS or not?. Crit Care.

[69] Komorowski M, Celi LA, Badawi O, Gordon AC, Faisal AA (2018). The Artificial Intelligence Clinician learns optimal treatment strategies for sepsis in intensive care. Nat Med.

[70] Ong E, Wong MU, Huffman A, He Y (2020). COVID-19 coronavirus vaccine design using reverse vaccinology and machine learning. bioRxiv.

[71] Adly AS, Adly AS, Adly MS (2020). Approaches Based on Artificial Intelligence and the Internet of Intelligent Things to Prevent the Spread of COVID-19: Scoping Review. J Med Internet Res.

